# Improved Localization of Insulinomas Using ^68^Ga-NODAGA-Exendin-4 PET/CT

**DOI:** 10.2967/jnumed.124.268158

**Published:** 2024-12

**Authors:** Marti Boss, Olof Eriksson, Kirsi Mikkola, Annemarie Eek, Maarten Brom, Mijke Buitinga, Adrienne H. Brouwers, Irina Velikyan, Beatrice Waser, Saila Kauhanen, Olof Solin, Camille Marciniak, Barbro Eriksson, Jean-Claude Reubi, Cyrielle Aveline, Damian Wild, Francois Pattou, Jean-Noel Talbot, Johannes Hofland, Anders Sundin, Pirjo Nuutila, John Hermans, Martin Gotthardt

**Affiliations:** 1 Department of Medical Imaging, Radboud University Medical Center, Nijmegen, The Netherlands;; 2 Department of Medicinal Chemistry, Science for Life Laboratory, Uppsala University, Uppsala, Sweden;; 3 Turku PET Center, University of Turku, Turku, Finland;; 4 Department of Nuclear Medicine, University Medical Center Groningen, Groningen, The Netherlands;; 5 Pathology Länggasse, Bern, Switzerland;; 6 Department of Gastrosurgery, Turku University Hospital, Turku, Finland;; 7 Department of General and Endocrine Surgery, University Hospital Lille, Lille, France;; 8 Section for Endocrine Oncology, Department of Medical Sciences, Uppsala University, Uppsala, Sweden;; 9 Institute of Pathology, Cell Biology and Experimental Cancer Research, University of Bern, Bern, Switzerland;; 10 Department of Nuclear Medicine, Hôpital Tenon, AP-HP, Paris, France;; 11 Division of Nuclear Medicine, University Hospital Basel, Basel, Switzerland;; 12 Center for Neuroendocrine and Endocrine Tumors, University Hospital Basel, Basel, Switzerland;; 13 Section of Endocrinology, Department of Internal Medicine, Erasmus Medical Center, Rotterdam, The Netherlands;; 14 Department of Surgical Sciences, Radiology and Molecular Imaging, Uppsala University, Uppsala, Sweden; and; 15 Department of Endocrinology, Turku University Hospital, Turku, Finland

**Keywords:** insulinoma, exendin PET/CT, diagnostic imaging, GLP-1 receptor

## Abstract

Precise anatomic localization of insulinomas is crucial for surgical treatment. Current routine noninvasive imaging techniques, including CT, MRI, and ^68^Ga-DOTA-somatostatin analog (DOTA-SSA) PET/CT, have limited sensitivity. Endoscopic ultrasound is highly sensitive but invasive. In this prospective multicenter study, we compared the diagnostic accuracy of ^68^Ga-NODAGA-exendin-4 (exendin) PET/CT with all routine imaging procedures for the localization of insulinomas. **Methods:** Sixty-nine adults with biochemically proven adult endogenous hyperinsulinemic hypoglycemia underwent exendin PET/CT and current routine imaging. Images were evaluated in a clinical reading and in an expert reading. Image quality was determined by quantitative analysis. **Results:** Based on clinical readings, the accuracy of exendin PET/CT (94.4%; 95% CI, 84.6%–98.8%) was greater than that of DOTA-SSA PET/CT (64.8%; 95% CI, 50.6%–77.3%), contrast-enhanced CT/contrast-enhanced diffusion-weighted imaging-MRI (83.3%; 95% CI, 70.7%–92.1%), and endoscopic ultrasound (82.8%; 95% CI, 64.1%–94.1%). In 13% of patients, a correct diagnosis was only reached after exendin PET/CT. Interobserver agreement between readings was higher for exendin PET/CT than for DOTA-SSA PET/CT and contrast-enhanced CT/contrast-enhanced diffusion-weighted imaging-MRI (Cohen κ, 1.0 vs. 0.5 and 0.55). Exendin PET/CT provided a higher insulinoma-to-background ratio (15.3 ± 6.7 vs. 5.2 ± 3.0) and contrast-to-noise ratio (22.6 ± 11.1 vs. 5.1 ± 3.7) than did DOTA-SSA PET/CT. **Conclusion:** This study demonstrates the superiority of exendin PET/CT in a unique prospective comparison to all current routine imaging modalities for preoperative localization of benign insulinomas, providing the level of evidence needed for clinical implementation.

## Visual Abstract

**Figure:**
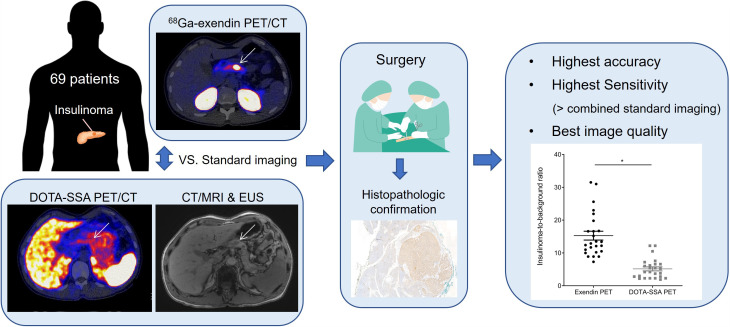

In this multicenter prospective trial, we assessed the diagnostic accuracy of ^68^Ga-NODAGA-exendin-4 for noninvasive localization of insulinomas. Insulinomas are neuroendocrine tumors arising from the pancreatic β-cells. With an incidence of 1–4 people per million per year, insulinomas are the most common cause of adult endogenous hyperinsulinemic hypoglycemia (AHH). Insulinomas cause episodic hypoglycemia, resulting in symptoms such as confusion, diplopia, dizziness and, in cases of prolonged hypoglycemia, even seizures, loss of consciousness, or death (1,2). AHH diagnosis is established biochemically by a positive prolonged fasting test, but localizing insulinomas for curative surgery planning relies on imaging.

Insulinomas are benign in about 90% of cases, and preferred surgical procedures are therefore pancreas-preserving, such as limited resection or enucleation (3), for which precise preoperative localization is essential to determine the anatomic relation to important structures such as the pancreatic duct or blood vessels. However, localization by imaging may be challenging because of their usually small size (82% are <2 cm, 47% are <1 cm). The noninvasive imaging methods of CT and MRI currently reach sensitivities of up to 70% and 90%, respectively (4), but despite the achieved progress, localizing insulinomas remains a challenge that often can only be solved by invasive procedures. Endoscopic ultrasound (EUS) has sensitivities for insulinoma detection reaching 75% (5). Another invasive technique is angiography with intraarterial calcium stimulation, so-called arterious stimulation venous sampling, with a sensitivity of about 85% (6), which only provides information about the part of the pancreas in which the insulinoma is located. Both EUS and arterious stimulation venous sampling are cumbersome for patients because of their invasive nature and the need for sedation. Also, they are not universally available, and their success is operator- and center-dependent.

PET imaging of the somatostatin receptor (SSTR) is the current routine nuclear imaging technique for localization of low-grade neuroendocrine tumors. With this technique, detection rates for insulinomas between 33% and 85% are reported, which are probably dependent on their (generally small) size and (often low) SSTR expression (7–9). Certain centers routinely perform ^18^F-3,4-dihydroxyphenylalanine (^18^F-DOPA) PET/CT for insulinoma detection, although the reported detection rates are inconsistent and based on limited patient numbers (7,10–12). Therefore, improved imaging procedures are required. Benign insulinomas express high levels of the glucagon-like peptide 1 receptor (GLP-1R) in 92% of cases (9), which therefore presents an attractive alternative target for nuclear imaging of insulinomas.

Here, we target the GLP-1R using ^68^Ga-labeled exendin-4, a stable analog of glucagon-like peptide 1, which selectively binds the GLP-1R with high affinity. Previous clinical studies have shown the potential of ^111^In-labeled exendin for insulinoma detection with SPECT (13–15). Subsequently, PET using ^68^Ga-labeled exendin (^68^Ga-DOTA-exendin-4), which has the combined benefit of a higher spatial resolution of PET, as compared with SPECT, and lower radiation burden to the patient, was shown to outperform ^111^In-labeled exendin (7,16).

In this study, we used an exendin-based radiotracer containing the chelator NODAGA, instead of DOTA, which enabled radiolabeling with ^68^Ga with a higher specific activity (17), enabling bolus administration of lower (sub)pharmacologic amounts of peptide to the patients, which could prevent or reduced adverse agonist effects such as hypoglycemia and nausea. In this prospective trial, we assessed the accuracy of the improved ^68^Ga-NODAGA-exendin-4 (exendin) PET/CT for localization of insulinomas in patients with proven AHH and compared that accuracy with all current routine noninvasive imaging procedures (contrast-enhanced [CE] CT or CE-diffusion-weighted imaging [DWI]-MRI and PET/CT with ^68^Ga-DOTATOC or ^68^Ga-DOTATATE (DOTA-somatostatin analog [SSA] PET/CT) and EUS. To the best of our knowledge, this is the first prospective clinical trial directly comparing all these imaging modalities.

## MATERIALS AND METHODS

### Study Design and Patients

In this prospective, multicenter study, patients were consecutively included at the Radboud University Medical Center Nijmegen and the University Medical Center Groningen in The Netherlands, the University Hospital of Turku in Finland, Uppsala University Hospital in Sweden, and Assistance Publique Hôpitaux de Paris in France. In total, 69 patients were included. Recruitment of patients was performed both directly in these participating centers as well as by referral from several other academic and tertiary hospitals across Europe. Patients with biochemically proven AHH with neuroglycopenic symptoms during the fasting test with low plasma glucose levels (<2.5 mmol/L), inappropriately high serum insulin (≥6 mU/L) and C-peptide levels (≥200 pmol/L), and relief of symptoms after glucose administration (Whipple triad) were enrolled. Exclusion criteria were evidence of malignancies other than insulin-producing tumors on CECT/CE-DWI-MRI, renal insufficiency (creatinine clearance, <40 mL/min), pregnancy, and breast feeding. The study was approved by the local institutional review boards of all participating institutes. All included patients provided written informed consent in accordance with provisions of the Declaration of Helsinki.

### Imaging Procedures

Imaging was performed according to local guidelines for insulinoma detection at each center, but certain requirements had to be fulfilled. For each patient, this included at least DOTA-SSA PET/CT, performed according to the European Association for Nuclear Medicine guidelines (18), and triple-phase CECT or CE-DWI-MRI. EUS was performed in a subgroup of patients when noninvasive imaging led to inconclusive results or it was deemed clinically relevant for other reasons. ^18^F-DOPA PET/CT was only performed in 1 center (details on CECT, CE-DWI-MRI, and ^18^F-DOPA PET/CT procedures are provided in the supplemental materials, available at http://jnm.snmjournals.org).

For exendin PET/CT, patients were injected intravenously with exendin (105.6 ± 2.3 MBq) as a slow bolus over 1 min. At 1 h and 2 h after tracer injection, PET/CT of the abdomen was acquired for 10 min per bed position in 2 bed positions. Further details, including the radiopharmaceutical preparation, are provided in the supplemental materials (19).

### Evaluation

The reference standard was histologic evaluation and clinical outcome. A favorable clinical outcome was defined as normalization of plasma glucose levels after surgery, improvement of quality of life, and reduction in fear of hypoglycemia, assessed using the hypoglycemia fear survey and SF36v2 health survey at an 8-wk follow-up. Clinical reports of all routine imaging procedures were provided by nonmasked radiologists and nuclear medicine physicians at the referring centers. Clinical reporting of 1 h and 2 h exendin PET/CT was performed locally by nuclear medicine physicians. In addition, all images were reevaluated (expert reading) by an experienced pancreas radiologist (with more than 20 y of experience) (CECT and CE-DWI-MRI) and a nuclear medicine physician (with more than 20 y of experience in neuroendocrine tumor imaging) (DOTA-SSA PET/CT and exendin PET/CT), who were masked to all clinical information (except for the AHH diagnosis) and all other imaging procedures. EUS results were not reevaluated because of the dynamic nature of the procedure. All EUS procedures were performed by experienced gastroenterologists in experienced centers according to the local guidelines.

When DOTA-SSA PET/CT and exendin PET/CT both identified a single insulinoma, subsequently confirmed on histopathology, quantitative PET analysis was performed by a nonmasked expert. The procedure for PET quantification is provided in the supplemental materials.

### Ex Vivo Tissue Analysis

Sections of tumor tissue from 24 patients were available to assess GLP-1R and SSTR subtype 2 expression using immunohistochemistry. Details of the immunohistochemistry protocol are provided in the supplemental materials.

### Statistical Analysis

Positive imaging results for insulinoma supported by histopathology and clinical follow-up after surgical resection were regarded as true positive findings. A patient-based analysis was performed (in patients with multiple lesions, an imaging result was regarded as a true positive if at least 1 histopathologically confirmed insulinoma was detected). We calculated 95% CIs for sensitivity using the Clopper–Pearson method. Numbers of correct assessments (localization in correspondence with histopathology) and incorrect assessments (localization not corresponding with histopathology) of all imaging procedures were represented in a 2 × 2 contingency table and were compared using a McNemar test for comparison of paired nominal data. Interobserver variation was calculated using Cohen κ. The insulinoma-to-background ratio and contrast-to-noise ratio of exendin PET/CT and DOTA-SSA PET/CT were compared using paired-sample *t* tests. All statistical analyses were performed using SPSS version 22 (IBM). A *P* value of less than 0.05 was considered significant.

## RESULTS

### Patients and Imaging

Baseline characteristics of the patients are in Supplemental Tables 1 and 2. Median time between DOTA-SSA PET/CT and exendin PET/CT was 1.0 wk (interquartile range, 0.1–3.0 wk), between CECT/CE-DWI-MRI and exendin PET/CT was 4.8 wk (interquartile range, 0.3–11.5 wk), and between EUS and exendin PET/CT was 4.4 wk (interquartile range, 1.9–14.7 wk).

Lesions suspicious for insulinoma were found on CECT in 63% of patients, on CE-DWI-MRI in 60% of patients, on CECT and CE-DWI-MRI combined in 68% of patients, on DOTA-SSA PET/CT in 55% of patients, on EUS in 71% of patients, and on exendin PET/CT in 79% of patients (Supplemental Table 1).

### Adverse Effects

Two patients (3%) experienced nausea, and 1 patient (1%) experienced vomiting after injection of exendin. No other adverse effects were observed. In all patients, exogenous glucose infusion was started as needed before or immediately after tracer injection. As a result, severe hypoglycemic episodes (blood glucose < 2.5 mmol/L) occurred in only 1 patient, a male patient requiring continuous daily intravenous infusions of 10% glucose infusions. He was given continuous infusion of 10% glucose during the examination, which was sped up after tracer injection combined with repeated injections of 30% glucose.

### Surgery and Histopathology

Results of imaging and surgical outcomes of all patients are provided in Supplemental Table 2. Results of all available imaging modalities were used for the surgical planning. Median time between exendin PET/CT and surgery was 7 wk (interquartile range, 4.6–10.7 wk). The study profile is in Supplemental Figure 1. Combined results of all imaging modalities revealed a suspicious lesion in 59 patients. Fifty-three patients underwent surgery, and histologic evaluation showed the presence of 1 or multiple insulinomas with a size of 7–27 mm. Plasma glucose values normalized after surgery with no further hypoglycemia at 2-wk follow-up, and patients showed improvement in quality of life and reduction in fear of hypoglycemia at 8-wk follow-up.

Of the 10 patients in whom all imaging failed, only 1 patient underwent surgery, which confirmed diffuse β-cell hyperplasia.

### Diagnostic Performance

Diagnostic performance was calculated in the patients who underwent surgery in whom a pathologic confirmation was available. Results of CECT, CE-DWIMRI, CECT with CE-DWI-MRI, DOTA-SSA PET/CT, EUS, and exendin PET/CT are summarized in Table 1. Based on the clinical reading, exendin PET/CT has higher accuracy and sensitivity (94.4% and 94.3%, respectively) than do DOTA-SSA PET/CT (64.8% and 64.2%, respectively), CE-DWI-MRI (73.0% and 72.2%, respectively), CECT (73.2.6%), CECT and CE-DWI-MRI combined (83.3% and 83.0%, respectively), and EUS (82.8% and 82.1%, respectively).

**TABLE 1.** Overview of Imaging Results in Participants Who Underwent Surgery with Conclusive Histopathologic Insulinoma Diagnosis

Parameter	Exendin PET/CT	DOTA-SSA PET/CT	CE-DWI-MRI	CECT	CECT/CE-DWI-MRI	EUS	^18^F-DOPA PET/CT
Insulinomas detected (clinical reading)	50/53 (94%)	35/53 (66%)	26/36 (72%)	30/40 (75%)	43/53 (81%)	24/28 (86%)	3/8 (38%)
Accuracy							
Based on clinical reading	94.4% (84.6%–98.8%)	64.8% (50.6%–77.3%)	73.0% (55.9%–86.2%)		83.3% (70.7%–92.1%)	82.8% (64.2%–94.1%)	
Based on study reading (blinded)	94.4% (84.4%–98.8%)	75.9% (62.4%–86.5%)	75.7% (58.8%–88.2%)		85.2% (72.9%–93.4%)		
Sensitivity							
Based on clinical reading	94.3% (84.3%–98.8%)	64.2% (49.8%–76.9%)	72.2% (54.8%–85.8%)	73.2% (57.1%–85.8%)	83.0% (70.2%–91.9%)	82.1% (63.1%–93.9%)	33% (7.5%–70.1%)
Based on study reading (blinded)	94.3% (84.3%–98.8%)	75.5% (61.7%–86.2%)	75.0% (57.8%–87.9%)	73.2% (57.1%–85.8%)	83.0% (70.2%–92.0%)		
Insulinoma-to-background ratio	15.3 ± 6.7	5.3 ± 3.0					
Contrast-to noise ratio	22.6 ± 11.1	5.1 ± 3.7					

Accuracy and sensitivity are given in percentages, and 95% CIs are in parentheses. Insulinoma-to-background ratios and contrast-to-noise ratios of tracer uptake are given as mean ± SD.

Cross tables comparing exendin PET/CT with the other imaging modalities, on the basis of the number of correct (corresponding with histopathology) and incorrect (not corresponding with histopathology) imaging results, are depicted in Table 2. A detailed explanation is given in the supplemental materials. The direct value of exendin PET/CT is demonstrated by the fact that in 7 of the 54 patients (13%) an insulinoma was correctly identified only with exendin PET/CT. When including EUS, an insulinoma was only found with exendin PET/CT in 5 of the 54 patients (9%). In these patients, the decision to perform surgery was based solely on the results of the exendin PET/CT.

**TABLE 2.** Contingency Table

	Exendin PET/CT	
Modality	Correct	Incorrect
DOTA-SSA PET/CT		
Correct	34	1
Incorrect	17	2
CECT/CE-DWI-MRI		
Correct	43	2
Incorrect	8	1
Routine noninvasive		
Correct	44	2
Incorrect	7	1
EUS		
Correct	22	2
Incorrect	5	0
Routine including EUS		
Correct	46	3
Incorrect	5	0

Number of correct (correctly corresponding to histopathology) and incorrect (not corresponding with histopathology) assessments of exendin PET/CT and DOTA-SSA PET/CT, CECT/CE-DWI-MRI, combination of all noninvasive routine imaging modalities (DOTA-SSA PET/CT, CE-DWI-MRI, and CECT), exendin PET/CT and EUS, and exendin PET/CT and combination of all routine imaging modalities including EUS.

### Interobserver Correlation

Image reevaluation by an expert observer increased the sensitivity of DOTA-SSA PET/CT from 64.2% to 75.5% and the sensitivity of CE-DWI-MRI from 72.2% to 75.0%. For the other imaging modalities, sensitivities based on the expert reading did not differ from the clinical reading (Table 1). Interobserver agreement between the readings was higher for exendin PET/CT (Cohen κ, 1.0) than for all other imaging modalities: DOTA-SSA PET/CT (Cohen κ, 0.50), CECT (Cohen κ, 0.50), CE-DWI-MRI (Cohen κ, 0.81), and CECT and CE-DWI-MRI combined (Cohen κ, 0.55). There was complete agreement for exendin PET/CT and strong agreement for CE-DWI-MRI (Cohen s κ between 0.8 and 0.90), and for DOTA, CECT, and CECT/CE-DWI-MRI, the level of agreement was weak (Cohen κ, <0.6).

### Quantitative Analysis

**FIGURE 1.**
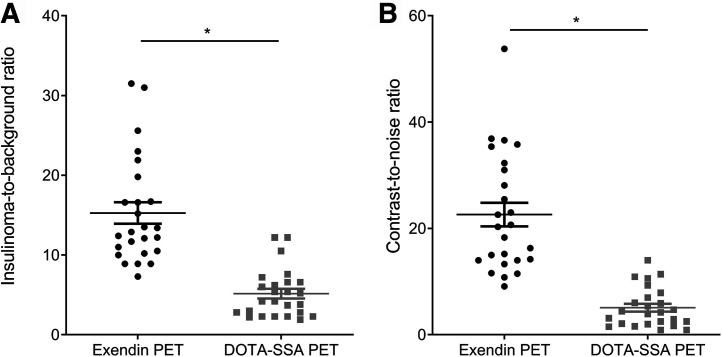
Insulinoma-to-background ratio and contrast-to-noise ratio of exendin PET/CT and DOTA-SSA PET/CT images. *P < 0.05.

**FIGURE 2.**
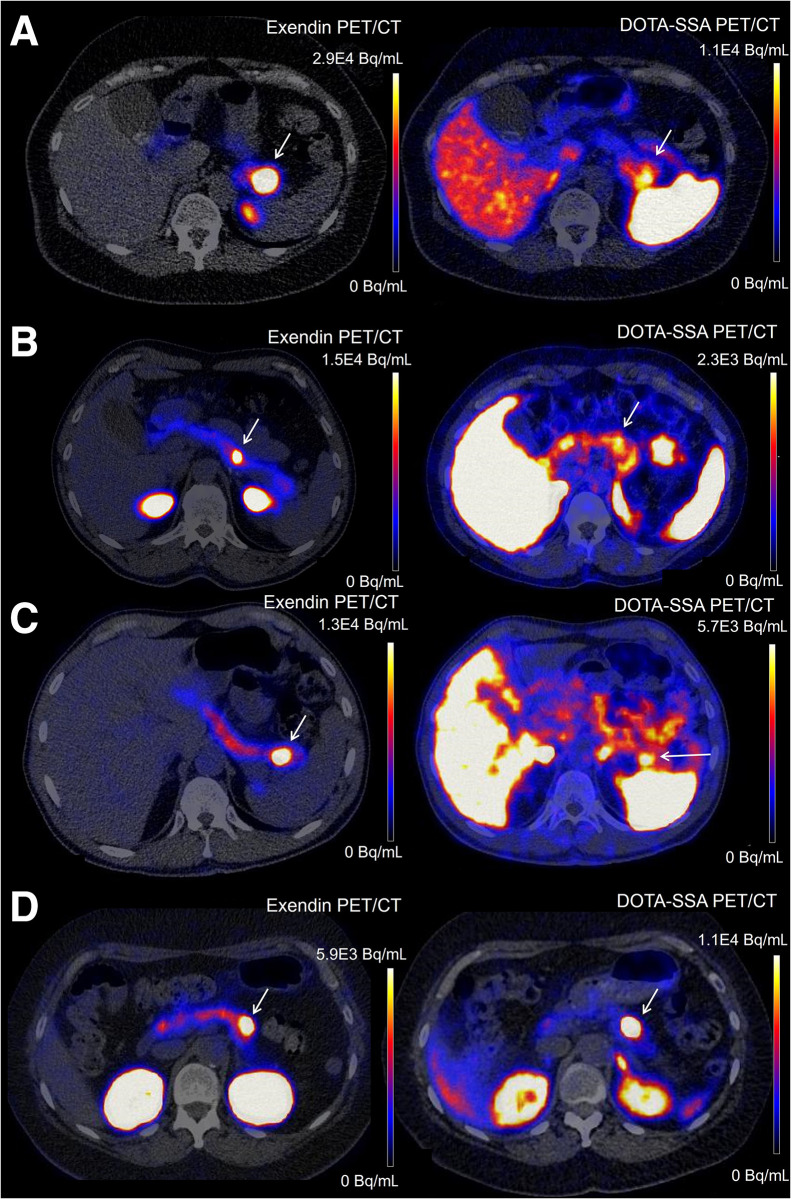
(A) Exendin PET/CT and DOTA-SSA PET/CT images of patient 56, with highest insulinoma-to-background ratio in exendin PET (31.5 vs. 2.3 in DOTA-SSA PET). Images of patients 4 (B) and 17 (C) show intermediate insulinoma-to-background ratios in exendin PET (8.9 and 10.4, respectively, vs. 2.2 and 2.3 in DOTA-SSA PET, respectively). (D) Images of patient 26 have lowest insulinoma-to-background ratio in exendin PET (7.3 vs. 12.2 in DOTA-SSA PET), showing still excellent visibility of insulinoma. Location of insulinomas is indicated with arrows.

Results of image quantification are shown in Figure 1. The mean ± SD insulinoma-to-background ratio was 15.3 ± 6.7 for exendin PET/CT and 5.2 ± 3.0 for DOTA-SSA PET/CT (*P* < 0.0001), and the mean ± SD contrast-to-noise ratio was 22.6 ± 11.1 for exendin PET/CT and 5.1 ± 3.7 for DOTA-SSA PET/CT (*P* < 0.0001). Examples of exendin PET/CT images from highest to lowest insulinoma-to-background ratio are depicted in Figure 2, illustrating the superior image quality of exendin PET/CT and the clear visibility of insulinomas, even in the case with the lowest insulinoma-to-background ratio.

### Ex Vivo Tissue Analysis

GLP-1R expression was found in all 24 analyzed resected lesions. Expression of SSTR subtype 2 was found in only 9 of 24 (38%) of these tumors. All these results are in line with diagnostic imaging, with all 24 lesions being detected using exendin PET/CT and only the 9 lesions showing SSTR subtype 2 expression being detected using SSTR-SSA PET/CT.

## DISCUSSION

This prospective multicenter study provides the first direct comparison of exendin PET/CT to all current routine noninvasive imaging methods, including DOTA-SSA PET/CT and EUS. The results show that exendin PET/CT is an excellent tool for the detection and localization of insulinomas, providing higher accuracy (94.4%) than DOTA-SSA PET/CT, CECT, CE-DWI-MRI, and even the more invasive EUS method. In 5 of 54 patients (9%), it was the only modality to provide correct detection.

Although previous studies on exendin PET/CT insulinoma detection have mostly focused on comparison to 1 specific routine imaging modality, this study provides a complete picture of the excellent diagnostic performance of exendin PET/CT.

DOTA-SSA PET was reported in a previous study (in 13 patients) to have an 85% detection rate (8). The current trial showed a lower detection rate (66%), which was confirmed by immunohistochemistry on ex vivo tissue samples, revealing GLP-1R expression in all lesions and SSTR subtype 2 expression only in 9 lesions that were detected with SSTR-SSA PET/CT. This result is in line with the previously reported low SSTR subtype 2 expression in insulinomas using in vitro autoradiography (9).

Concerning the comparison to CE-DWI-MRI, our results are in line with the prospective study by Antwi et al., who reported 93.9% accuracy with exendin PET/CT in the detection of insulinomas and 67.6% accuracy with CE-DWI-MRI (7). In the current trial, exendin PET/CT was compared with CECT, CE-DWI-MRI, or the combination of CECT and CE-DWI-MRI, performed by the referring centers, reflecting the standard of care of patients with confirmed AHH and, thus, the current best clinical practice, which achieved a combined 83.3% accuracy (CECT and CE-DWI-MRI). This somewhat better accuracy compared with CE-DWI-MRI alone in the study of Antwi et al. is probably due to the high rate of combined CE-DWI-MRI and CECT (in ∼40% of patients) and the larger median size of insulinomas (15 mm vs. 12 mm).

Because of the invasive nature of EUS, it was not performed as a standard procedure in all participants but was performed in a subgroup of 34 patients on clinical grounds. The overall sensitivity of exendin PET/CT (94.3%) in these patients was higher than the sensitivity of EUS (82.1%). In 27 of 34 patients (79%), exendin PET/CT as a primary modality would have localized the insulinoma, rendering an invasive EUS procedure redundant, reducing the burden of the diagnostic process for the patients.

Although ^18^F-DOPA PET/CT was only performed in 9 patients in this study, the low detection rate of 33% combined with previous inconsistent results for insulinoma detection with ^18^F-DOPA (7,10–12) point toward the superiority of exendin PET/CT, which detected all insulinomas in these 9 patients.

The excellent sensitivity of the exendin PET/CT is well illustrated by the 15 mm (range, 7–27 mm) median histopathology-based size of the insulinomas identified by exendin PET/CT, including 5 insulinomas smaller than 10 mm. We experienced 3 false-negative exendin PET/CT findings, corresponding to 2 somewhat larger insulinomas: 20 mm (patient 24) and 25 mm (patient 61) and 1 small insulinoma of 12 mm (patient 42). These false negatives are therefore probably related to a lack of GLP-1R expression, which is in agreement with the 92% expression rate of GLP-1R that was found by Reubi et al. (9).

**FIGURE 3.**
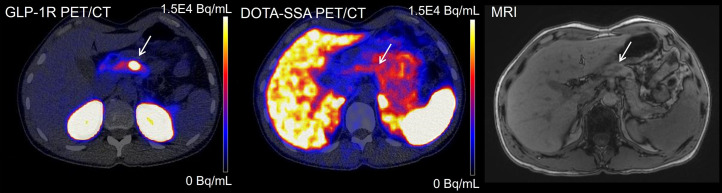
Images of exendin PET/CT, DOTA-SSA-PET/CT, and CE-DWI-MRI (T1-weighted precontrast) of patient 8. Location of insulinoma in pancreas body is indicated with arrows. Only exendin PET/CT was true positive in this patient.

Besides the high sensitivity of exendin PET/CT, we also observed strikingly superior interobserver agreement between readings of exendin PET/CT and the other techniques. This can be explained by the excellent image quality of exendin PET/CT with very high insulinoma-to-background ratios, enabling unambiguous detection and localization of insulinomas. An interesting example is patient 8, in whom both CE-DWI-MRI and DOTA-SSA PET/CT were negative. Subsequent exendin PET/CT showed a distinct focal uptake in the pancreatic body. When CE-DWI-MRI was reassessed and correlated with the exendin PET/CT, a corresponding hypointense lesion was identified (Fig. 3).

In contrast to previous studies on exendin PET/CT in patients with AHH (7,16,20), we used the chelator NODAGA instead of DOTA. Because of the higher specific activity, we injected a peptide dose of 4–7 μg instead of the 12–24 μg used with ^68^Ga-DOTA-exendin. This resulted in the occurrence of nausea in only 3% of patients, as compared with 27% of patients with ^68^Ga-DOTA-exendin (7). The ability to perform exendin PET/CT with a low peptide dose of ^68^Ga-NODAGA-exendin is also favorable for use in children, as shown previously in a prospective trial (21).

The correct diagnosis in 51 of 54 patients indicates that exendin PET/CT could suffice as the primary diagnostic imaging method, potentially as a one-stop-shop procedure combined with CECT for optimal anatomic correlation and surgical planning (the combination providing correct diagnosis in 53 of 54 patients in this study). This would thus considerably reduce the imaging work-up, eliminating the need for other noninvasive or invasive diagnostic procedures in the vast majority of patients. This would reduce the burden for patients and could help to substantially lower the costs for the care of patients with AHH.

Until now, there has been insufficient data to get this imaging method into the guidelines. Several retrospective studies and 2 prospective trials with limited patient numbers (Supplemental Table 3) have been reported (16,22–25), which offer a lower level of evidence than our prospective trial. There have been 2 previous prospective cohort studies with considerable patient numbers (52 patients in both cases) (7,26), which fail to provide a full comparison of exendin PET to all current routine methods for insulinoma detection. The full comparison with current routine techniques with a high level of evidence of this study can finally pave the way toward incorporation of exendin PET in the guidelines for insulinoma diagnostics.

A potential limitation of this study is the comparison of exendin PET/CT to routine procedures performed according to the protocols of the referring centers, with potential technical variations. However, we believe that this reflects the current real-world clinical practice in insulinoma imaging and thus improves the reliability of our data.

The study is furthermore limited by the fact that biochemically proven AHH is no confirmation of the presence of an insulinoma, since symptoms could be caused by diffuse β-cell hyperplasia. Therefore, only patients undergoing surgery could be included in the analysis, and false and true negatives cannot be assessed. Although insulinomas are the most common cause of AHH, in the general population, approximately 0.5% to 5% of cases are instead caused by diffuse β-cell hyperplasia. The percentage of patients with this diagnosis in this study is potentially higher because of an inclusion bias, explained by the referral of patients with negative localization results on CECT/CE-DWI-MRI from nonstudy centers.

## CONCLUSION

This study demonstrates the superior performance of exendin PET/CT in a first-time direct prospective comparison to all noninvasive imaging modalities which encompass the current clinic practice for preoperative localization of benign insulinomas as well as the more invasive EUS. Besides the higher detection rate of insulinomas, the excellent image quality of exendin PET/CT leads to a complete interobserver agreement. On top of that, use of the improved NODAGA-exendin decreased the side effects of the tracer as compared with earlier studies. Because of the high sensitivity and excellent imaging quality shown here, exendin PET/CT should be considered as a potential primary diagnostic imaging modality in patients with AHH. Exendin PET/CT combined with CECT could provide a one-stop-shop procedure for insulinoma localization, greatly reducing and simplifying the imaging work-up for the large majority of patients.

## DISCLOSURE

The research leading to these results has received funding from the European Community’s Seventh Framework Programme (FP7/2014-2018) under grant agreement no. 602812 (BetaCure). Olof Eriksson is an employee of Antaros Medical AB and cofounder of Antaros Tracer AB. No other potential conflict of interest relevant to this article was reported.

## Notes

KEY POINTS **QUESTION:** Does exendin PET/CT improve the noninvasive localization of insulinomas? **PERTINENT FINDINGS:** This prospective clinical trial, comparing exendin PET/CT to all current routine imaging modalities, shows an improved detection rate of insulinomas with excellent image quality. **IMPLICATIONS FOR PATIENT CARE:** Exendin PET/CT could greatly reduce and simplify the imaging work-up for the large majority of insulinoma patients.
